# Meyer's loop tractography for image-guided surgery depends on imaging protocol and hardware

**DOI:** 10.1016/j.nicl.2018.08.021

**Published:** 2018-08-13

**Authors:** Maxime Chamberland, Chantal M.W. Tax, Derek K. Jones

**Affiliations:** a Cardiff University Brain Research Imaging Centre (CUBRIC), School of Psychology, Cardiff University, Cardiff, United Kingdom; b School of Psychology, Faculty of Health Sciences, Australian Catholic University, Victoria, Australia

## Abstract

**Introduction:**

Surgical resection is an effective treatment for temporal lobe epilepsy but can result in visual field defects. This could be minimized if surgeons knew the exact location of the anterior part of the optic radiation (OR), the Meyer's loop. To this end, there is increasing prevalence of image-guided surgery using diffusion MRI tractography. Despite considerable effort in developing analysis methods, a wide discrepancy in Meyer's loop reconstructions is observed in the literature. Moreover, the impact of differences in image acquisition on Meyer's loop tractography remains unclear. Here, while employing the same state-of-the-art analysis protocol, we explored the extent to which variance in data acquisition leads to variance in OR reconstruction.

**Methods:**

Diffusion MRI data were acquired for the same thirteen healthy subjects using standard and state-of-the-art protocols on three scanners with different maximum gradient amplitudes (MGA): Siemens Connectom (MGA = 300 mT/m); Siemens Prisma (MGA = 80 mT/m) and GE Excite-HD (MGA = 40 mT/m). Meyer's loop was reconstructed on all subjects and its distance to the temporal pole (ML-TP) was compared across protocols.

**Results:**

A significant effect of data acquisition on the ML-TP distance was observed between protocols (*p* < .01 to 0.0001). The biggest inter-acquisition discrepancy for the same subject across different protocols was 16.5 mm (mean: 9.4 mm, range: 3.7–16.5 mm).

**Conclusion:**

We showed that variance in data acquisition leads to substantive variance in OR tractography. This has direct implications for neurosurgical planning, where part of the OR is at risk due to an under-estimation of its location using conventional acquisition protocols.

## Highlights

• Diffusion MRI data from 13 subjects on 3 scanners with different maximum gradient amplitude.
• Optic radiation tractography was performed using “standard” and “state-of-the-art” acquisitions.
• Meyer's loop to Temporal Pole distance varies up to 16.5 mm for the same subject across protocols.
• State-of-the-art protocols provide Meyer's loop measurements that are in line with ex-vivo studies.
• Surgical planning for anterior temporal lobe resection depends on protocol and available hardware.

## Graphical abstract

**Figure:**
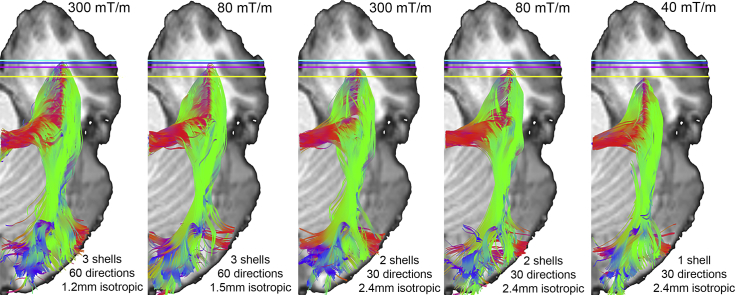

## 1 Introduction

Epilepsy is a neurological disorder characterised by recurrent seizures. Temporal lobe epilepsy (TLE) seizures originate in the temporal lobe and may be focal, localised to discrete cortical or subcortical regions within the temporal lobe, or may spread to other parts of the brain. An effective treatment for TLE consists of performing anterior temporal lobe resection (ATLR), often combined with amygdalohippocampectomy (Wiebe et al., 2001). One approach, the trans-middle temporal gyrus approach (or transcortical), enters through the trans-middle temporal gyrus. A second approach, the sub-temporal approach, is less complex but can cause significant damage to the neocortex. A third, and most technically difficult approach, is one where the neurosurgeon has to access the brain though the transsylvian fissure. This technique reduces displacement of brain tissues but carries a greater risk of vascular complications (Bandt et al., 2013; Kovanda et al., 2014).

**Fig. 1:**
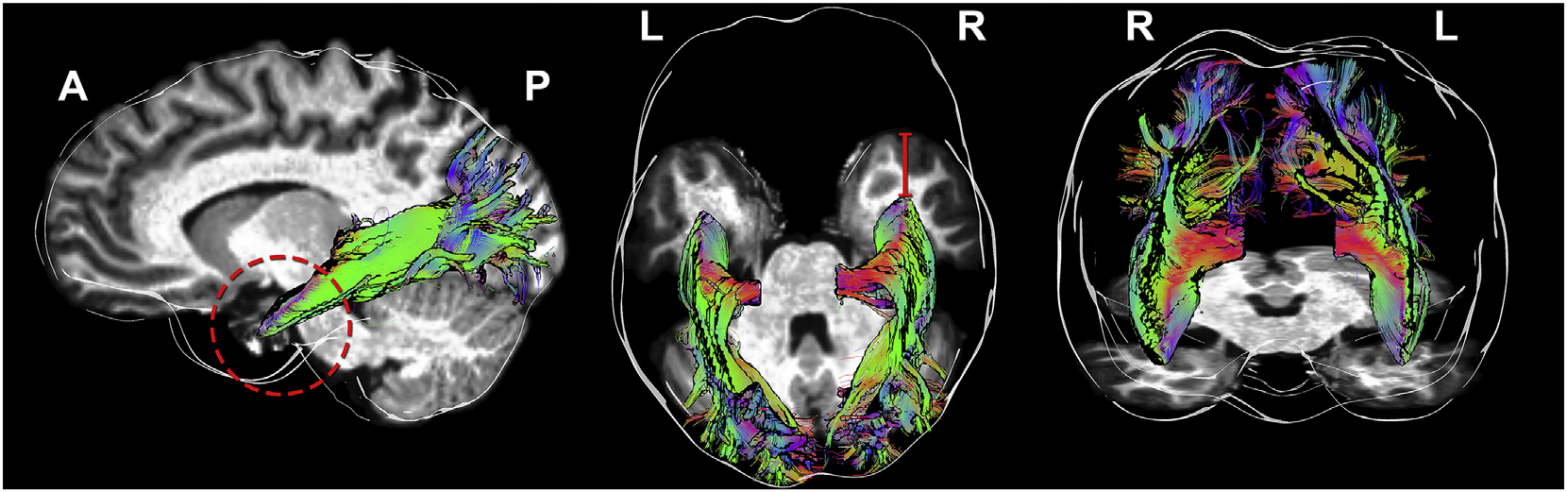
Tractography of the optic radiation. Streamlines are colour coded by orientation (i.e., left-right: red, antero-posterior: green, superior-inferior: blue). Meyer's loop is formed of streamlines sharply bending in the temporal lobe (left panel, dashed area). The red line (middle panel) shows the distance from Meyer's loop to the temporal pole (ML-TP). Viewing angles: lateral, superior and frontal, respectively.

All three surgical approaches can result in visual field deficits (VFDs) in more than half of the patients undergoing the procedures, reducing the quality of life of these patients (Pathak-Ray et al., 2002). This is due to the transection of the optic radiation (OR), a white matter (WM) fibre bundle of utmost importance, responsible for transmitting visual information between the lateral geniculate nucleus (LGN) and the visual cortex (Ebeling and Reulen, 1988; Rubino et al., 2005; Winston et al., 2012). Meyer's loop is a section of the OR that projects most anteriorly, with a sharp backwards bend (Chamberland et al., 2017; Ebeling and Reulen, 1988; Goga and Ture, 2015). The extent and angulation to which the Meyer's loop fans out anteriorly is known to vary between subjects. From a neurosurgical perspective, it behooves us to pay particular attention to the 3D trajectory of the OR in order to reduce the risk of inducing VFDs. Knowing the exact location of the OR, and more specifically of the Meyer's loop, is crucial information that may help reduce morbidity for patients undergoing ATLR. Therefore, subject-specific information on the distance between the most anterior tip of the Meyer's loop and the temporal pole (TP), also referred to as the ML-TP distance (Fig. 1, Table 1), could greatly aid surgeons in preparation for such intervention, and lead to improved patient outcome. However, the anterior extent of Meyer's loop is 1) often located within the resection area; 2) known to vary between subjects (Goga and Ture, 2015) and 3) invisible on conventional MRI techniques.

**Table 1:** Average ML-TP distance derived from ex-vivo dissection studies.

Authors	Mean ML-TP + range (mm)
Ebeling and Reulen (1988)	27 (22–37)
Peuskens et al. (2004)	27 (15–30)
Rubino et al. (2005)	25 (22−30)
Choi et al. (2006)	31.4 (28–34)
Chowdhury and Khan (2010)	26 (23−31)

Since the OR cannot be identified visually during surgery, substantial effort has been exerted in obtaining an accurate localization of the OR using tractography derived from dMRI (for review, see (Benjamin et al., 2014; Lilja and Nilsson, 2015; Mandelstam, 2012)). Yet, various issues hamper the complete and accurate reconstruction of the human OR, particularly due to its characteristic high curvature (Fig. 1). A common observation is that the anterior extent of the Meyer's loop is underestimated by most reconstruction methods currently employed in clinical research (Lilja et al., 2014). Indeed, an accurate Meyer's loop tractography should produce a dense set of streamlines (Benjamin et al., 2014; Goga and Ture, 2015) with no separation or gaps between the fibres (Fig. 1, red circle), mirroring the known anatomy of the bundle (Ebeling and Reulen, 1988; Goga and Ture, 2015). A possible limitation comes from the inability of the diffusion tensor (DT) representation, which is often employed in clinical research, to resolve the underlying complex local directions of the WM fibre pathways (Tournier et al., 2011).

In addition, the impact of image acquisition on Meyer's loop reconstruction remains unclear. Conventional whole-brain clinical dMRI acquisitions aim to reduce scan duration by limiting the number of diffusion encoding directions (e.g., between 12 and 60 at a single b-value) and spatial resolution (e.g., at best 2 × 2 × 2 mm^3^ isotropic). From an acquisition point-of-view, imaging data with higher spatial resolution seems promising for capturing the intricacies and fine sharp turn of the Meyer's loop. However, this often comes at a cost of reduced signal-to-noise ratio (SNR), which can be compensated for by increasing the acquisition time. However, recent advances in hardware have led to dramatic improvements in data quality and reductions in scan duration (Sotiropoulos et al., 2013). Stronger magnetic field gradients not only allow higher SNR at high b-values, but also provide better spatial resolution. Concomitantly, achieving better SNR and/or angular resolution allows better estimation of complex fibre orientations (Sotiropoulos and Zalesky, 2017; Tournier et al., 2011). In addition, multiple b-values allow more accurate estimation of partial volume effects, a necessity for achieving better estimation of WM directions (Jeurissen et al., 2014), which in turn will directly impact the results of tractography (Maier-Hein et al., 2017).

In this paper, we hypothesize that a more complete Meyer's loop reconstruction can be achieved by using state-of-the-art (SoA) hardware and analysis techniques. To this end, we compare the ML-TP distance derived from tractography of the same thirteen subjects (healthy controls) acquired on three different scanners, using a mixture of five standard (Std) and SoA protocols.

## 2 Methods

### 2.1 Data acquisition

Written informed consent was given by all subjects. Meyer's loop reconstructions were evaluated on a dedicated dataset (Tax et al., 2018) of the same 13 subjects (labelled from A to M) acquired on three different 3 T scanners with different maximum gradient amplitudes (MGA): Siemens Connectom (MGA = 300 mT/m); Siemens Prisma (MGA = 80 mT/m) and GE Excite-HD (MGA = 40 mT/m) using Std and SoA acquisition protocols, with the latter having higher spatial and angular resolution. Acquisition parameters for all protocols are summarised in Table 2. A T_1_-weighted 1 mm isotropic MPRAGE image was acquired for each subject and scanner for anatomical reference.

**Table 2:** Acquisition parameters for each protocol. Std: standard. SoA: state-of-the-art. †: cardiac gated.

Parameters	Std-300 mT/m	SoA-300 mT/m	Std-80 mT/m	SoA-80 mT/m	Std-40 mT/m
Scanner	Siemens Connectom	Siemens Connectom	Siemens Prisma	Siemens Prisma	GE Excite-HD
Resolution (mm^3^)	2.4 × 2.4 × 2.4	1.2 × 1.2 × 1.2	2.4 × 2.4 × 2.4	1.5 × 1.5 × 1.5	2.4 × 2.4 × 2.4
Directions (per b-value)	30	60	30	60	30
b-values (s/mm^2^)	1200 3000	1200 3000 5000	1200 3000	1200 3000 5000	1200
TE/TR (ms)	89/7200	68/5400	89/7200	80/4500	89/†

### 2.2 Data pre-processing

Eddy current distortion- and motion correction was performed using FSL EDDY (Andersson and Sotiropoulos, 2016) (fsl.fmrib.ox.ac.uk). Susceptibility distortions were also corrected using FSL TOPUP (Andersson et al., 2003) for both the Connectom and Prisma data. The Connectom data were additionally corrected for geometric distortions due to gradient non-linearity (Glasser et al., 2013). All data were then upsampled to 1 × 1 × 1 mm^3^ and aligned between scanners, using the Prisma standard data set as the reference frame for each subject.

This step facilitated the positioning of region-of-interests (ROIs), reducing subjective bias at the inter-individual level and allowed a direct comparison of tractography results between protocols. More specifically, the Connectom and Prisma data were affinely co-registered using the mean b = 0 and b = 1200 s/mm^2^ images using ANTs (Avants et al., 2011), followed by appropriate B-matrix rotation (Leemans and Jones, 2009). Since the GE Excite-HD data were acquired without reverse phase encoding, images were non-linearly warped to the reference space to correct for susceptibility distortions.

### 2.3 Local modelling and tractography

**Fig. 2:**
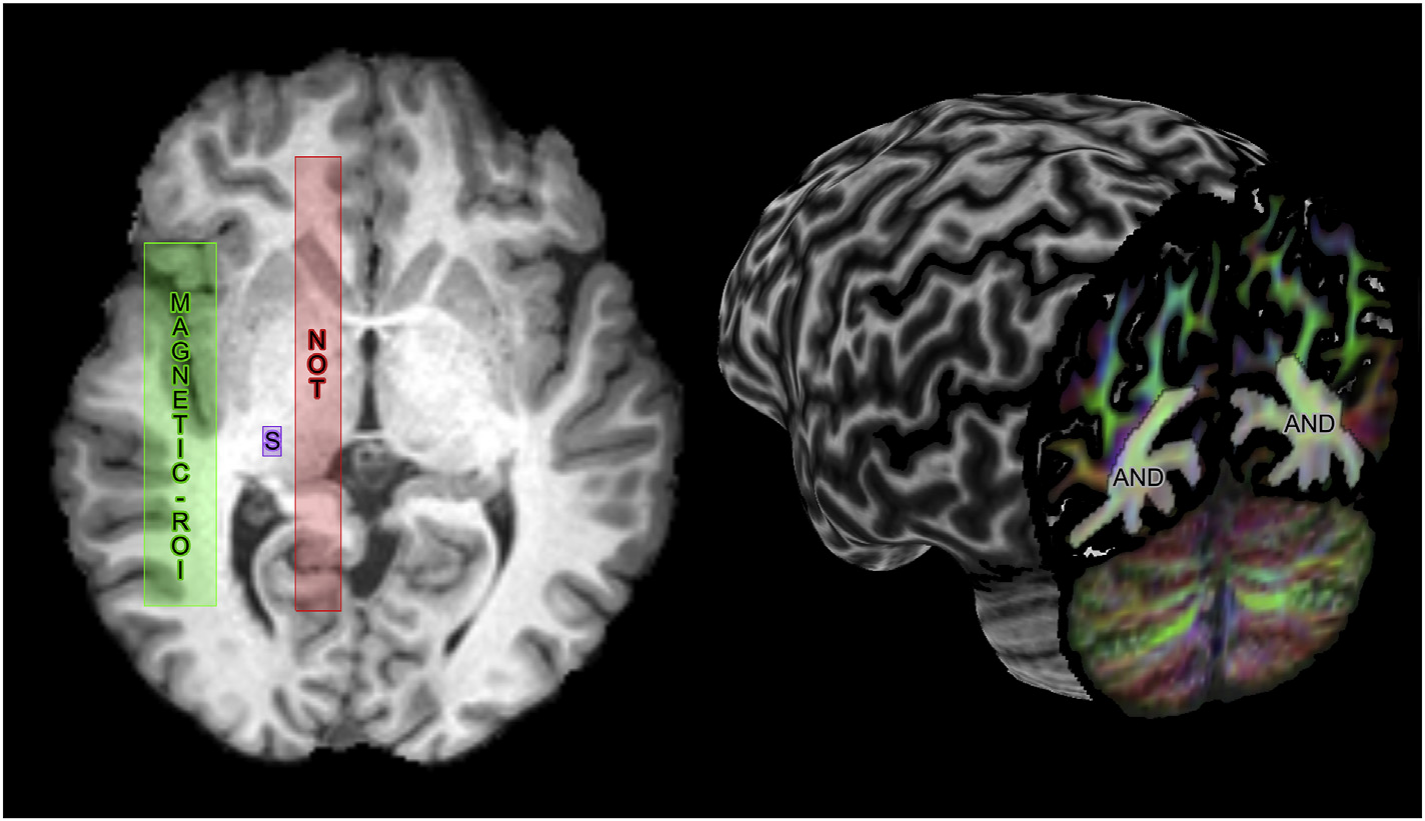
ROI positioning for each subject. A cubic seeding ROI (purple, S) was positioned in the LGN area. An include region (AND, independently displayed for both hemispheres) was placed in the sagittal stratum. An exclude ROI (red, NOT) was also placed lateral to the midsagittal plane. Finally, a magnetic-ROI (green) with anatomical prior pointing towards the occipital pole was placed in Meyer's loop area (Chamberland et al., 2017).

Next, fibre orientation distribution functions (fODFs) were derived using multi-shell multi-tissue constrained spherical deconvolution (MSMT-CSD) (Jeurissen et al., 2014), for both Connectom and Prisma data. For the single b-value Std-40 mT/m data, free-water elimination was performed by only supplying the WM and cerebro-spinal fluid response functions to the MSMT-CSD algorithm in MRtrix. The resulting fODF peaks (thresholded at amplitudes ≥0.1) were used to perform tractography using the FiberNavigator (Chamberland et al., 2014). Tractography parameters were fixed for all subjects and protocols (angular threshold: 45^°^, step size: 1 mm, min/max length: 30/200 mm). For both hemispheres of each subject, a 5 × 5 × 5 mm^3^ seeding ROI with 8000 seeds was interactively placed anterolaterally to the LGN (Chamberland et al., 2017; Martinez Heras et al., 2015), with initial propagation direction oriented along the left/right axis (Fig. 2, purple). The seed-ROI was large enough to capture part of the LGN, but also interfaced with the adjacent WM to ensure that the first few critical tractography steps where performed on well-defined diffusion orientations (Chamberland et al., 2017; Martinez Heras et al., 2015; Sherbondy et al., 2008). An inclusion planar-ROI centered in the sagittal stratum (Fig. 2, AND) and an exclusion sagittal plane (Fig. 2, NOT) acted as filtering regions to reduce false-positives.

Next, a 16 × 80 × 40 mm^3^ *magnetic*-ROI (Chamberland et al., 2017) was placed in the anterior temporal lobe (Fig. 2, green) where the Meyer's loop typically undertakes its turnaround. As we previously demonstrated (Chamberland et al., 2017), this new ROI-mechanism aims to facilitate Meyer's loop delineation by incorporating anatomical priors of the expected fibre orientation to the tractography algorithm. The magnetic-ROI not only selects streamlines that reach it, but also facilitate the choice of direction to follow based on a user-defined propagation direction. In the case of Meyer's loop, streamlines that exit the LGN and enter that ROI will propagate along the fODF direction that points towards the occipital pole instead of any other existing choice. Finally, the resulting streamlines were quality controlled visually for all subjects to ensure that no spurious or isolated streamline remained.

### 2.4 Statistical analysis

The ML-TP distance was measured using the axial projection of the most anterior part of the Meyer's loop (i.e., the absolute Y-component of the 3D Euclidean distance). This distance was then normalized by head size using the most posterior point of each occipital pole (OP) (i.e., 100%·(ML-TP)/(TP-OP)), allowing for inter-individual comparisons. Unless specified, all other instances of this measurement within this paper refer to the raw ML-TP distance. A one-way ANOVA was conducted to compare the effect of scanning protocol on the normalized ML-TP distance. *Post-hoc* results were corrected for multiple comparisons using the Bonferroni-Holm test. Finally, a lateralization index was derived for all sixty-five pairs of Meyer's loop reconstructions by subtracting measurements of the right hemisphere from those of the left one. A negative value indicates a shorter ML-TP on the left side (e.g., larger anterior extent of the Meyer's loop).

## 3 Results

**Fig. 3:**
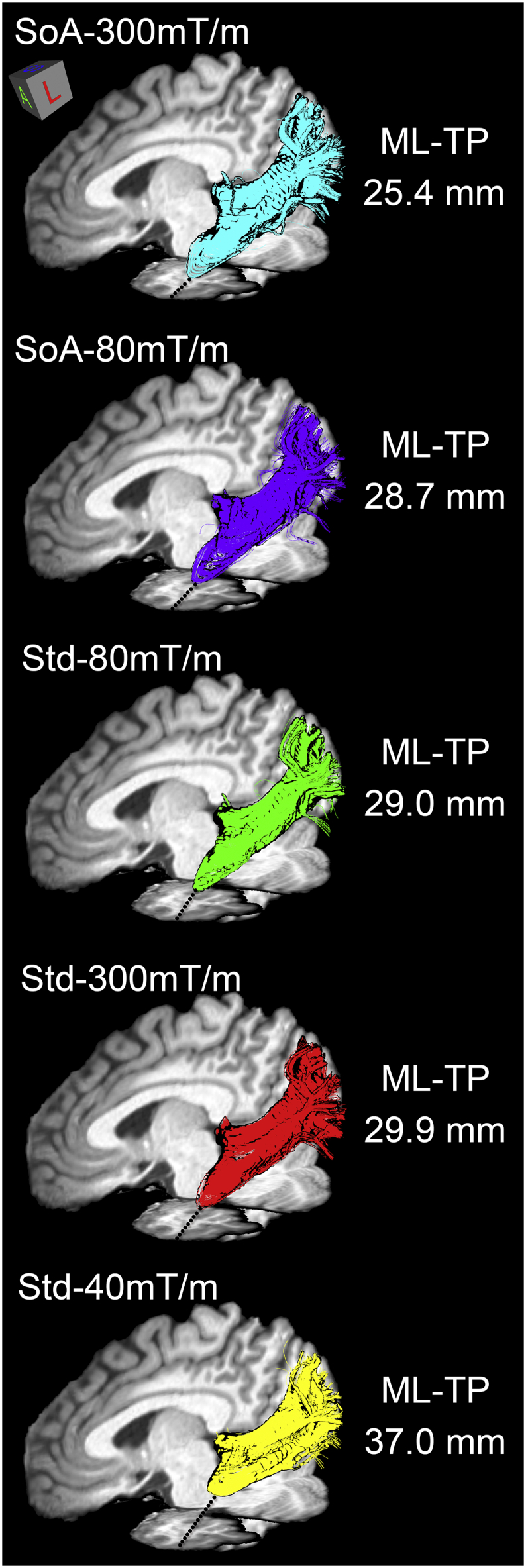
Qualitative visualization of Meyer's loop for a single subject's hemisphere (subject H) across each protocol. In a direct comparison, one can observe a larger anterior extent of the optic radiation in SoA protocols (blue and purple) when compared to Std protocols (red, green and yellow). View: oblique sagittal.

The tract reconstructions were highly consistent with anatomical descriptions of the OR for all subjects. A representative reconstructed OR of a single subject across all protocols is illustrated in Fig. 3, using an oblique lateral view. In a direct side-by-side comparison, one can observe a larger anterior extent of the OR for the SoA protocols (e.g., blue and purple) when compared to data from the Std protocols. More importantly, even though the ML-TP distances are larger in the Std protocols, the tractography result from all data sets show dense reconstructions of streamlines in Meyer's loop, with no separation between the streamlines. Coloured lines are drawn in the image space to better depict the intra-subject variance across protocols.

**Fig. 4:**
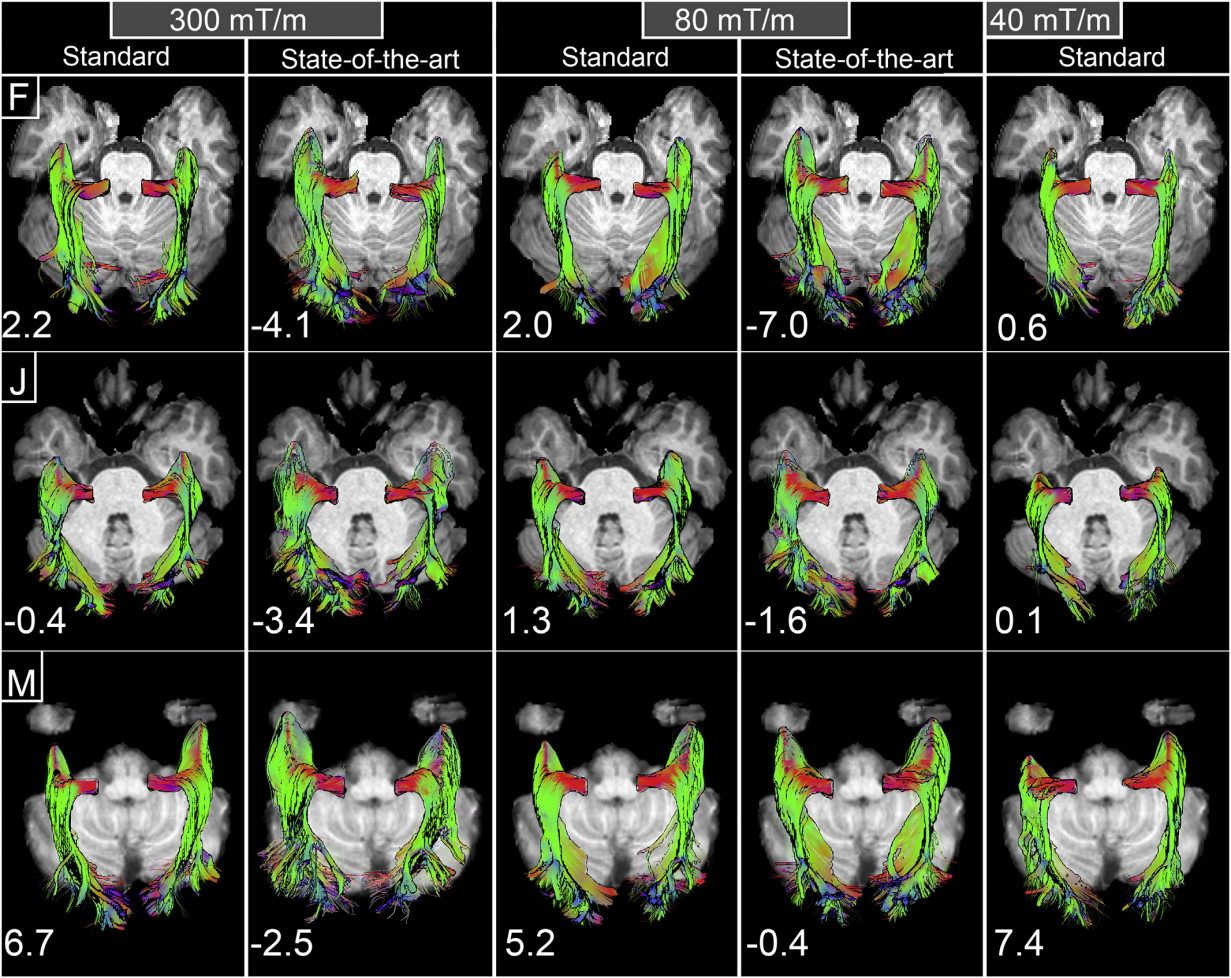
Qualitative and quantitative asymmetry of reconstructed Meyer's loop illustrated for 3 representative subjects (subjects F, J, M). Each row represents the same subject across different protocols. A trend towards a left-lateralization of the Meyer's loop is mostly observed in SoA reconstructions. Annotated numbers on the lower left hand corners refer to the lateralization indices.

Fig. 4 illustrates the asymmetry of the Meyer's loop reconstruction for 3 representative subjects, both qualitatively and quantitatively. In this figure, each row represents the same subject across different protocols. The axial planes were set so that the entire OR was visible (e.g., positioned below the deepest point of the Meyers's loop in the Z axis). Intra- and inter- subject variability of the anterior extent of the Meyer's loop is revealed by the lateralization indices. A trend towards a left-lateralization of the Meyer's loop is observed in SoA reconstructions (see Table 3).

**Table 3:** Average raw ML-TP distance and lateralization index (LI) across all protocols. The shortest distances were measured in SoA acquisitions. In addition, both SoA protocols revealed a left lateralization trend. However, only the 300mTm/m one was significant (*: *p* < .05).

Protocol	Mean ML-TP (left)	Mean ML-TP (right)	Mean LI
SoA-300 mT/m	25.0 (19.9–28.9)	26.6 (23.1–29.8)	−1.6 (*)
SoA-80 mT/m	26.9 (23.5–29.9)	27.7 (23.9–34.0)	−0.8
Std-80 mT/m	30.4 (25.1–36.6)	30.1 (25.1–34.0)	0.4
Std-300 mT/m	31.4 (25.2–39.0)	30.7 (27.9–34.9)	0.7
Std-40 mT/m	35.4 (30.0–43.5)	35.0 (28.3–43)	0.4

Raw ML-TP distances and lateralization indices across all protocols are reported in Table 3. At the group level, SoA protocols reconstruct Meyer's loop with a smaller ML-TP distance. Most notable are the SoA-300 mT/m results with a mean ML-TP distance for the left and right hemispheres of 25.0 mm (range: 19.9–28.9 mm) and 26.6 mm (range: 23.1–29.8 mm), similar to the results obtained from direct measurement through ex vivo dissection (Table 1). On average, this places Meyer's loop 9.4 mm (range: 3.7–16.5 mm) in front of what was inferred by the Std-40 mT/m protocol. In addition, the SoA-300 mT/m protocol also showed a significant left lateralization of the OR (*p* < .05).

**Fig. 5:**
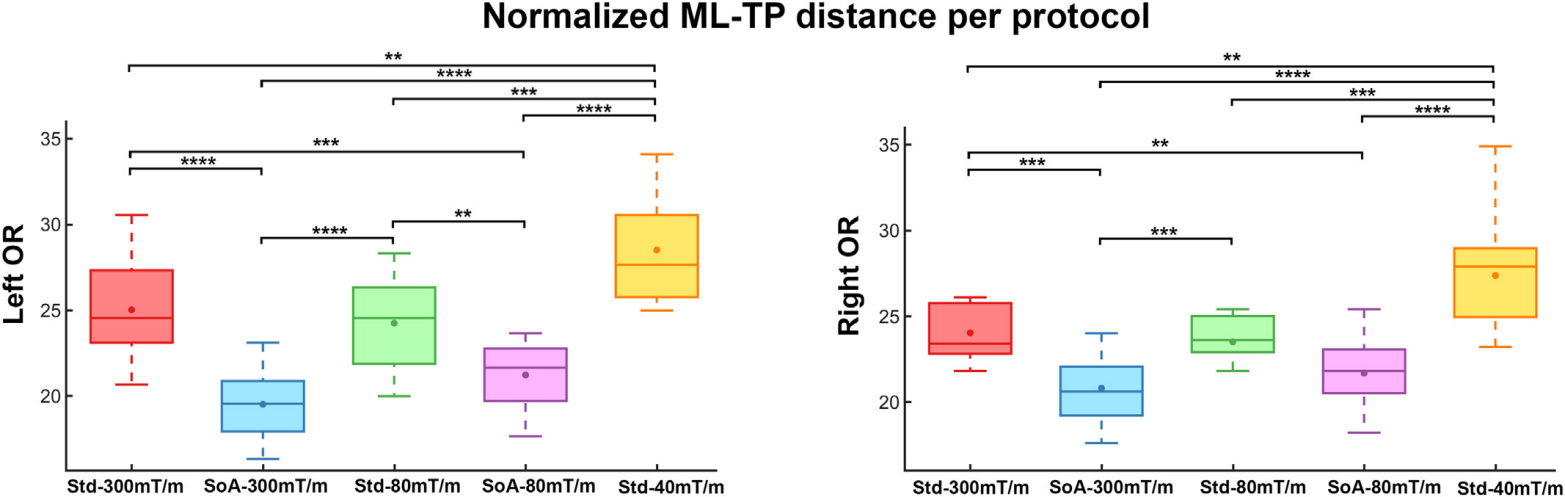
Quartile boxplots comparing the effect of scanning protocol on the reconstructed normalized ML-TP distances for both hemispheres. SoA protocols reconstruct a larger Meyer's loop anterior extent when compared to Std ones. **: *p* < .01 ***: *p* < .001 ****: *p* < .0001 (Bonferroni corrected). Dot: mean. Line: median. Note that measurements reported in this figure were normalized by the head size of each participant across all scanners.

The effect of scanning protocol on the measured ML-TP distances for both hemispheres (head normalized across scanners) is shown in Fig. 5. Based on an analysis of variance, a significant effect of scanning protocol on reconstructed ML-TP distance was found at the p < .05 level for both left [F(4, 60) = 25.05, *p* = 3.17E-12] and right hemispheres [F(4, 60) = 18.46, *p* = 6.16E-10]. Fig. 5 also indicates that i) the mean measurements of both SoA protocols (blue and pink) were significantly different than the Std-40 mT/m protocol (*p* < .0001); ii) both Connectom and Prisma Std protocols (red and green) indicate significant difference with the Std-40 mT/m one (*p* < .01 and 0.001). Additionally, both SoA reconstructions indicate significant differences over all Std reconstructions, with the exception of the right hemisphere SoA and Std 80 mT/m (Fig. 5, *p* = .02).

## 4 Discussion

### 4.1 Measurements and asymmetry of the Meyer's loop

**Fig. 6:**
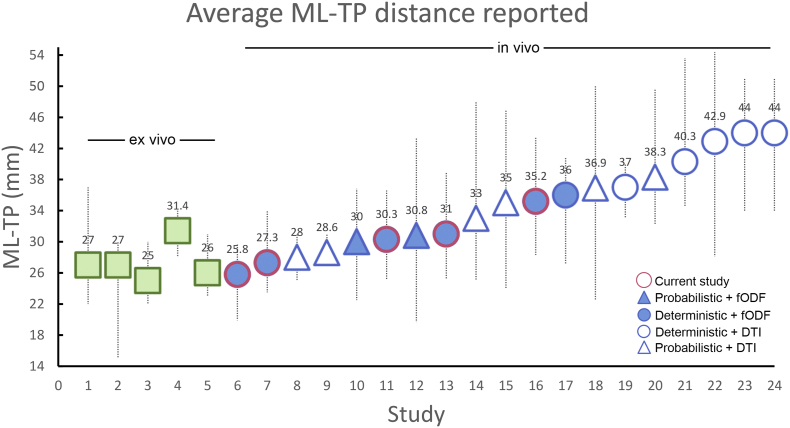
Average ML-TP distance reported in the literature for healthy controls (both hemispheres combined). 1: Ebeling and Reulen (1988). 2: Peuskens et al. (2004). 3: Rubino et al. (2005). 4: Choi et al. (2006). 5: Chowdhury and Khan (2010). 6: Current study (SoA-300 mT/m). 7: Current study (SoA-80 mT/m). 8: Sherbondy et al. (2008). 9: Tax et al. (2014). 10: Meesters et al. (2017). 11: Current study (Std-80 mT/m). 12: Kammen et al. (2016). 13: Current study (Std-300 mT/m). 14: Lilja et al. (2014). 15: Yogarajah et al. (2009). 16: Current study (Std-40 mT/m). 17: Chamberland et al. (2017). 18: Dayan et al. (2015). 19: Yamamoto et al. (2005). 20: James et al. (2015). 21: Wu et al. (2011). 22: de Gervai et al. (2014). 23: Nilsson et al. (2007). 24: Lilja et al. (2014).

In this work, we focused on the challenge of reconstructing the optic radiation, which is important for epilepsy surgery in the temporal lobe, by providing a quantitative comparison of several acquisition protocols from different scanners. There are two main findings associated with this study. First, a significantly reduced ML-TP distance was found for both SoA-300 mT/m and SoA-80 mT/m acquisitions, with a mean ML-TP distance across both hemispheres of 25.8 and 27.3 mm, respectively. These measurements are directly in the range of what has been reported by several ex vivo dissection studies (see Fig. 6). The Std-300 mT/m and Std-80 mT/m protocols followed closely with a mean ML-TP measurement of 31.0 and 30.3 mm, respectively. This finding is also supported by the average location of the OR across protocols, as shown in Supplementary Fig. 1 (red-yellow). Additionally, it is worth noting that, although underestimating the loop when compared to other protocols, the Std-40 mT/m measurements derived from single-shell CSD estimated a smaller ML-TP distance than most previously-published DTI studies (Figs. 6, 35.2 mm). We attribute this to the use of the MAGNET algorithm in the current study, which specifically addresses the high curvature of Meyer's loop (Chamberland et al., 2017). Interestingly, matching protocols on different scanners (i.e., Std-80 mT/m vs Std-300 mT/m and SoA-80 mT/m vs SoA-300 mT/m) did not yield any significant difference in terms of tractography reconstructions (Fig. 5). Yet, higher gradient strengths allow for better SNR per unit time for higher b-values, which in turn will allow more reliable estimation of fibre orientations.

The second finding is related to the asymmetry of the OR. A trend towards a left lateralization of the OR was found for both SoA protocols, although only significant for the 300 mT/m protocol. This is directly in line with other dissection and tractography studies that also observed a reduced ML-TP distance on the left hemisphere (de Gervai et al., 2014; James et al., 2015; Lilja et al., 2014; Mandelstam, 2012; Nowell et al., 2015; Yogarajah et al., 2009). In a recent study, a significant left lateralization of the Meyer's loop was also found in a cohort of 90 children, but only in male subjects (Dayan et al., 2015). Another group also found a leftward lateralization of the OR using data from the Human Connectom Project, but only in terms of volume (Kammen et al., 2016).

### 4.2 Pre-processing and tractography choices

Potential sources of error can include registration, interpolation and the tractography algorithm itself. To begin with, our choice of registering the pre-processed data to a single reference space could potentially induce errors in distance measures, especially if the registration fails to properly align the volumes. However, visual inspection of all datasets confirmed that good registration was achieved for all subjects. This was performed not only looking at the overlay of raw diffusion images, but also by inspecting the spatial organisation of the diffusion directions (i.e., fODF glyphs), with respect to the anatomy in all three planes. Moreover, performing the measurement comparisons in the native space of each acquisition might have introduced additional variance in the ML-TP distance, since the exact position of anatomical landmarks used for measurements cannot be guaranteed. In this work, we also chose to interpolate the diffusion data to the resolution of the anatomical data (i.e., 1 mm^3^) before modelling the fODFs. It has been shown recently that interpolating the raw diffusion volumes outperforms the direct interpolation of diffusion-derived measures (Dyrby et al., 2014).

Another point to consider following interpolation of the data is that data with different spatial resolutions have different partial volume effects. For most diffusion methods using a single b-value, it is a challenging task to correctly estimate the fODFs in the presence of cerebrospinal fluid (CSF), and our results derived from the Std-40 mT/m protocol may have been affected by this. Multi-shell modelling approaches can overcome these drawbacks at the tissue interfaces (Jeurissen et al., 2014). Additionally, the performance of multi-shell methods will typically increase with the total number of measurements acquired, leading to more accurate tractography (e.g., SoA datasets employed in this study). Finally, an inherent limitation to our study is that the true underlying fibre architecture of the Meyer's loop is unknown for each subject, and thus the true ML-TP distance is also unknown.

### 4.3 Neurosurgical implications

From a clinical perspective, obtaining a physically-defined border of a specific pathway (e.g., tip of Meyer's loop) is of main interest to minimize post-operative morbidity. Tractography is an invaluable tool for surgical planning, being the only tool available for neurosurgeons to visualize fibre pathways prior to surgery (Essayed et al., 2017; Nimsky et al., 2016) by super-imposing them onto surgical navigation scans. Multiple groups have attempted to model the OR using tractography for surgical planning (Borius et al., 2014; Chen et al., 2009; Lilja et al., 2015; Meesters et al., 2017; Nilsson et al., 2007; Nowell et al., 2015; Yogarajah et al., 2009), suggesting a direct link between the ML-TP distance and VFDs (Chen et al., 2009). A more recent clinical study revealed that patients with VFDs had their Meyer's loop estimates located anterior to the resection margins, whereas in patients without VFDs, the Meyer's loop reconstruction did not reach as far as the resection area (Winston et al., 2012). Keeping these results in mind, one can consider a thought experiment in which a surgeon is about to perform ATLR on a patient whose data were acquired with the 40 mT/m protocol described here (e.g., Fig. 3, right). In that case, not knowing that the anterior aspect of the Meyer's loop depicted by this Std protocol is 7 to 12 mm behind what is inferred from more advanced acquisitions can be dramatic for the patient's outcome. This undershoot in ML-TP may in part explain why more than half of ATLR patients suffer from VFDs post-operatively (Chen et al., 2009; Winston et al., 2012; Yogarajah et al., 2009).

In light of these results, there remains little standardisation in the acquisition and reconstruction of diffusion data for surgical planning. Indeed, application of Meyer's loop tractography in neurosurgery greatly relies on detailed anatomical knowledge and on the effects of different acquisitions and analysis methods. OR reconstructions derived from tractography should be carefully interpreted since the possible post-surgical outcome for patients will be dependent on the hardware and protocol that is available in the hospital. A similar study to the current one but based on a large cohort of patients would therefore be valuable for the neurosurgical community.

### 4.4 Recommendations and future directions

Despite being unable to recover multiple fibre orientations, the diffusion tensor remains the most widely employed representation for surgical planning. Based on our Meyer's loop tractography results, being able to resolve complex fibre architecture seems crucial for every pipeline that aims to reconstruct the full anterior extent of the OR. Without the ability to accurately recover complex fibre configurations, tractography-recovered streamlines are prone to a premature halt in the WM.

In recent years, more advanced approach have been proposed, surpassing the limitations of conventional DTI (Jeurissen et al., 2017; Tournier et al., 2011). MSMT-CSD is a promising new technique that provides sharper diffusion profiles, allowing the estimation of fODFs in complex regions. In addition, MSMT-CSD provides more precise WM fibre orientation estimates at the tissue interfaces, which is crucial for tracking to OR from the LGN. Although not focused on the Meyer's loop reconstruction, MSMT-CSD was also recently applied in a clinical context to reconstruct the entire optic pathway of twenty-six paediatric tumor patients (Hales et al., 2018). Sixty diffusion gradients distributed over two shells (i.e., b = 1000 and 2200 s/mm^2^) were used for a total scan duration of 7 min 50s, indicating that MSMT-CSD can be employed in a clinically feasible scan time.

Moreover, tractography methods are now at a stage where anatomical information can be introduced as input to help reduce false-positives and premature tract termination (for review, see (Jeurissen et al., 2017)). It is worth mentioning at this point that most of the aforementioned advanced tractography methods are already publicly available to clinicians via open-source software packages such as the FiberNavigator (chamber.github.io/fibernavigator_single), MRtrix (mrtrix.org) and Dipy (nipy.org/dipy).

For those with limited access to SoA hardware and acquisition protocols, alternative strategies can be considered. From an acquisition point of view, it is suggested to balance between spatial and angular resolution (Sotiropoulos and Zalesky, 2017), with the latter having a better impact on tractography (Vos et al., 2016). From an image processing point of view, latest advances in image quality transfer (Alexander et al., 2014) may also be considered. Methodological advances in denoising and upsampling approaches (Dyrby et al., 2014) also help to improve both spatial and angular resolution. Furthermore, microstructure-informed tractography (Girard et al., 2017) is a novel addition to conventional tracking based on the idea that each fibre bundle possess unique microstructural features (e.g., axon diameter). These features can then be used to guide streamline propagation and potentially solve reconstruction ambiguities in regions of complex fibre architecture. In this work, we addressed this problem by incorporating prior anatomical information. Encoding such external prior information to the tracking is in great part responsible for achieving a complete reconstruction of Meyer's loop (Chamberland et al., 2017). Using the MAGNET algorithm, streamlines are allowed to undertake within-voxel sharp turns as they enter the directionally-encoded ROI (Fig. 2, green); a scenario that is not supported by most other tractography algorithms.

## 5 Conclusion

Taken together, we demonstrate that (where exactly the same tractography parameters are used) the choice of acquisition protocol affects Meyer's loop reconstruction. Specifically, acquiring data with a higher spatial and angular resolution (Vos et al., 2016) with more b-values gives rise to a more complete anterior delineation of the Meyer's loop, assuming that SoA reconstructions inferred from tractography do reflect Meyer's loop true location. This has important applications in ATLR where surgeons may transect part of the Meyer's loop due to an under-estimation of its location. The results also underpin, when time permits, the importance of using SoA imaging protocols for neurosurgical planning.

## Appendix A Supplementary data

Supplementary material Image 1
