# Supplementary material for: Meyer's loop tractography for image-guided surgery depends on imaging protocol and hardware
Source: Neuroimage Clin. 2018 Aug 13;20:458–65. doi: 10.1016/j.nicl.2018.08.021 (PMC6096050; doi:10.1016/j.nicl.2018.08.021)
Supplement: Supplementary file 1 — Supplementary material [file mmc1.docx]

**Supplementary materials**

| Authors | Algorithm | Mean ML-TP + range (mm) | # of subjects |
| --- | --- | --- | --- |
| *Current study SoA-300 mT/m* | Det. + fODF | 25.8 (19.9 – 29.8) | 13 |
| *Current study SoA-80 mT/m* | Det. + fODF | 27.3 (23.5 – 34) | 13 |
| Sherbondy et al. (2008) | Prob. + DT | 28 (25 – 31) | 8 |
| Tax et al. (2014) | Prob. + DT | 28.6 (27 – 31) | 3 |
| Meesters et al. (2017) | Prob. + fODF | 30 (22.5 – 36.8) | 8 |
| *Current study Std-80 mT/m* | Det. + fODF | 30.3 (25.1 – 36.6) | 13 |
| Kammen et al. (2015) | Prob. + fODF | 30.8 (19.7 – 43.4) | 215 |
| Current study Std-300 mT/m | Det. + fODF | 31.0 (25.2 – 39.0) | 13 |
| Lilja et al. (2014) | Prob. + DT | 33 (25 – 48) | 11 |
| Yogarajah et al. (2009) | Prob. + DT | 35 (24 – 47) | 21 |
| *Current study Std-40 mT/m* | Det. + fODF | 35.2 (28.3 – 43.5) | 13 |
| Chamberland et al. (2017) | Det. + DT | 36 (27.1 – 40.8) | 15 |
| Dayan et al. (2015) | Prob. + DT | 36.9 (22.5 – 50) | 14 |
| Yamamoto et al. (2005) | Det. + DT | 37 (33 – 40) | 5 |
| James et al. (2015) | Prob. + DT | 38.3 (32.2 – 49.7) | 75 |
| Wu et al. (2011) | Det. + DT | 42.9 (28.1 – 54.4) | 10 |
| De Gervai et al. (2014) | Det. + DT | 44 (34 – 51) | 20 |
| Nilsson et al. (2007) | Det. + DT | 44 (34 – 51) | 7 |
| Lilja et al. (2014) | Det. + DT | 44 (34 – 51) | 11 |

Supplementary Table 1 caption: Average ML-TP distance from tractography studies performed on healthy controls (both hemispheres combined). Det.: Deterministic. Prob.: Probabilistic.


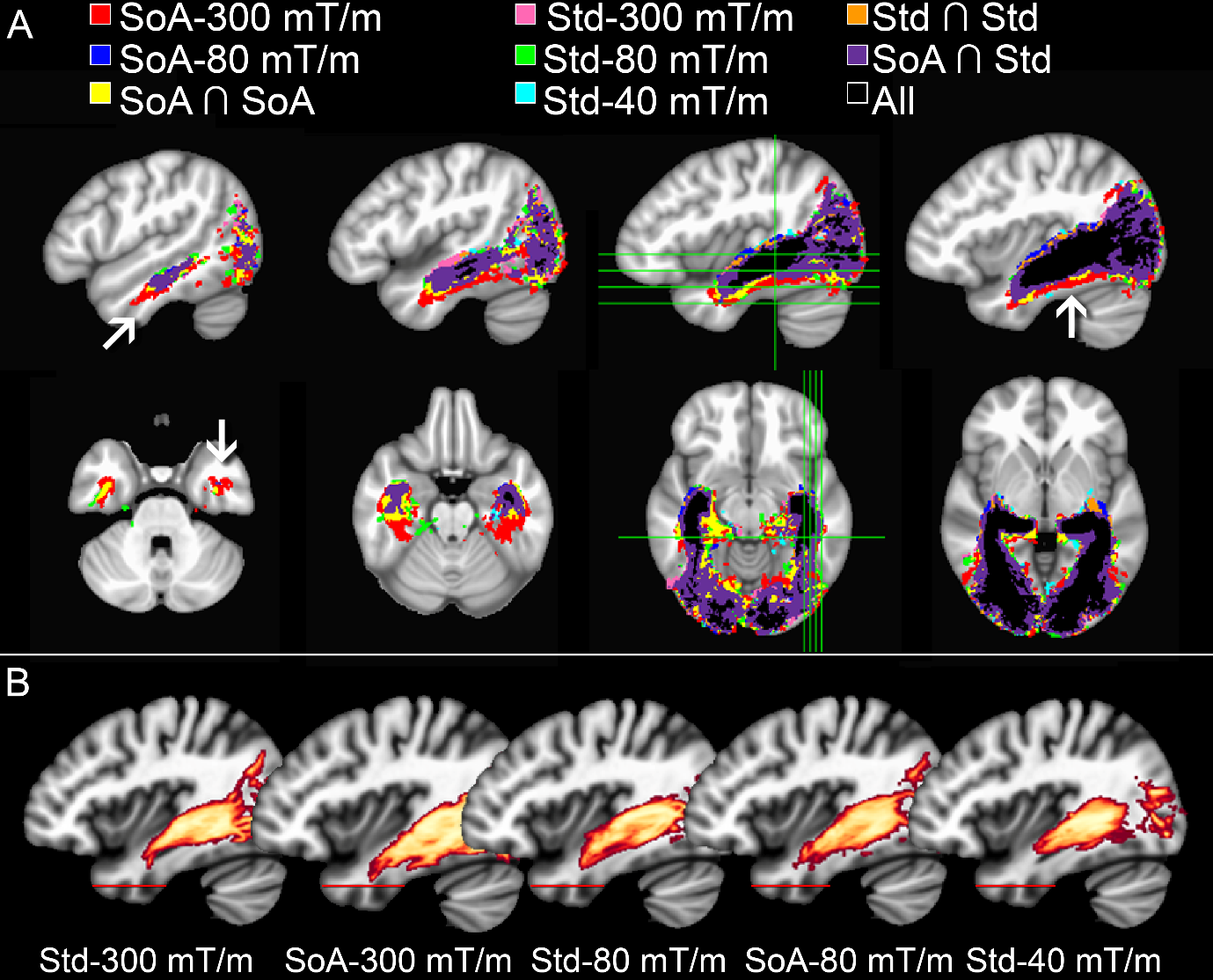


Supplementary Figure 1 caption: Spatial extent of the optic radiation illustrated using per-scanner group maps (thresholded at 95%). Binary colour encoding was applied to distinguish voxels where two or more protocols overlapped. One can observe that SoA-300 mT/m (red) shows a larger Meyer’s loop spatial extent at the anterior and ventral aspects of the bundle (arrows).
